# Cerebral Microcirculation during Experimental Normovolaemic Anemia

**DOI:** 10.3389/fneur.2016.00006

**Published:** 2016-02-02

**Authors:** Judith Bellapart, Kylie Cuthbertson, Kimble Dunster, Sara Diab, David G. Platts, O. Christopher Raffel, Levon Gabrielian, Adrian Barnett, Jenifer Paratz, Rob Boots, John F. Fraser

**Affiliations:** ^1^Department of Intensive Care, Royal Brisbane and Women’s Hospital, Herston, QLD, Australia; ^2^Critical Care Research Group, University of Queensland, St Lucia, QLD, Australia; ^3^Medical Engineering Research Facility, Queensland University of Technology, Brisbane, QLD, Australia; ^4^Department of Cardiology, The Prince Charles Hospital, Chermside, QLD, Australia; ^5^Medical Research Centre, Medical School, University of South Australia, Adelaide, SA, Australia; ^6^School of Public Health and Social Work, Institute of Health and Biomedical Innovation, Queensland University of Technology, Brisbane, QLD, Australia; ^7^Department of Intensive Care, The Prince Charles Hospital, Chermside, QLD, Australia

**Keywords:** anemia, APP staining, histology, microcirculation, microspheres

## Abstract

Anemia is accepted among critically ill patients as an alternative to elective blood transfusion. This practice has been extrapolated to head injury patients with only one study comparing the effects of mild anemia on neurological outcome. There are no studies quantifying microcirculation during anemia. Experimental studies suggest that anemia leads to cerebral hypoxia and increased rates of infarction, but the lack of clinical equipoise, when testing the cerebral effects of transfusion among critically injured patients, supports the need of experimental studies. The aim of this study was to quantify cerebral microcirculation and the potential presence of axonal damage in an experimental model exposed to normovolaemic anemia, with the intention of describing possible limitations within management practices in critically ill patients. Under non-recovered anesthesia, six Merino sheep were instrumented using an intracardiac transeptal catheter to inject coded microspheres into the left atrium to ensure systemic and non-chaotic distribution. Cytometric analyses quantified cerebral microcirculation at specific regions of the brain. Amyloid precursor protein staining was used as an indicator of axonal damage. Animals were exposed to normovolaemic anemia by blood extractions from the indwelling arterial catheter with simultaneous fluid replacement through a venous central catheter. Simultaneous data recording from cerebral tissue oxygenation, intracranial pressure, and cardiac output was monitored. A regression model was used to examine the effects of anemia on microcirculation with a mixed model to control for repeated measures. Homogeneous and normal cerebral microcirculation with no evidence of axonal damage was present in all cerebral regions, with no temporal variability, concluding that acute normovolaemic anemia does not result in short-term effects on cerebral microcirculation in the ovine brain.

## Introduction

Anemia and its pathophysiological implications have been investigated in recent years (1). The deleterious effects of blood transfusions have been well documented (2–4). Since the TRICC Trial (5), evidence based guidelines (6, 7) have influenced the use of blood transfusions, particularly in critically ill patients. However, a *post hoc* subgroup analysis of head injury patients from the TRICC trial (8) assessed the effects of restrictive transfusion strategies with hemoglobin concentrations maintained between 7.0 and 9.0 g/dL compared to the maintenance of hemoglobin concentrations between 10.0 and 12.0 g/dL on neurological outcome at 60 days post-injury. In that study, the authors found a significantly higher incidence of blood transfusions in the liberal group, with no differences in the ICU or 60-day mortality, length of stay, or organ failures. However, the direct effects of anemia on neurological outcomes were not assessed. It is still controversial whether anemia worsens brain injury (9–11). A recent study (12) examined the associations between the management of acute head injury patients with a restrictive versus a liberal transfusion threshold and the administration of erythropoietin with neurological outcomes at 6 months as the primary endpoint. No difference in neurological outcome was demonstrated with either intervention, with the lowest hemoglobin being 10 g/dL. However, several studies (13–18) have raised concerns regarding the safety of tolerating anemia in the setting of acute head injury. These studies showed that a sustained hemoglobin concentration of 7 g/dL correlated with secondary neurological injury from cerebral tissue hypoxia (13), with blood transfusion significantly increasing the partial pressure of cerebral tissue oxygenation (PTiO_2_) independently of cerebral perfusion pressure (14, 15). In addition, critical levels of PTiO_2_ (<15 mmHg) are correlated with a significant increase in the incidence of stroke and mortality (17–20). As a result of cerebral tissue ischemia, cerebral microdialysis markers, such as lactate/pyruvate ratio, glutamate, and glycerol, rise as a reflection of altered cellular metabolism (21, 22). In the absence of clinically available measures of cerebral microcirculation, transfusion thresholds in head injury patients may be better determined by physiological markers of cerebral ischemia and hypoperfusion rather than those recommended in more general critical care case mix where only mortality outcomes are assessed (22).

In light of this controversy, this novel study aimed to directly quantify regional microvascular blood flow (RMBF) using cytometric counting of color-coded microspheres with the potential neurological injury assessed using amyloid precursor protein (APP) staining of brain tissue of sheep exposed to normovolemic anemia.

## Materials and Methods

### Animal Care and Preparation

All experimental procedures were approved by the Animal Ethics Committee of the Queensland University of Technology and University of Queensland Committee and complied with local guidelines. Sheep were selected as the preferred animal model because of their cerebral anatomical similarities with humans, specifically within the gyrencephalic structure, allowing better examination of gray–white matter. In addition, the hemoglobin dissociation curve in sheep is comparable to that of human with a considerable amount of experimental studies in ovine neurosciences. A triple lumen central line (Cook Medical INC., QLD, Australia) and two 16-Fr introducer sheaths were placed in the right internal jugular (RIJ) vein in a convenience sample of six Merino sheep wethers weighing 65 ± 6.01 kg. Via the central line, the sheep were placed under general anesthesia with an initial bolus of 5 mg/kg ketamine and a maintenance infusion between 0.5 and 1 mg/kg/h. Sedation was achieved with a combined infusion of midazolam (0.5 mg/kg/h), fentanyl (10 mcg/kg/h), and alfaxalone (6 mg/kg/h). This is a common veterinarian anesthetic regimen that has stable cardiovascular effects. Hydration was maintained with an infusion of Hartmann’s solution at a rate up to 2 mL/kg/h titrated to a central venous pressure (CVP) of 6–10 mmHg. Cardiovascular monitoring included cardiac output and vascular resistances via a Swan–Ganz catheter, as in previous models (23). A 5-Fr umbilical vessel catheter (Argyle, Tyco HealthCare, Mansfield, MA, USA) was placed in the right femoral artery to allow for blood withdrawal at a rate of 10 mL/min. Orotracheal intubation was performed with a 10-mm endotracheal tube (SIMS Portex, UK). Sheep were ventilated at 12 breaths per minute with tidal volumes of 8 mL/kg and 5 cm H_2_O of PEEP with an initial FiO_2_ of 1.0. The FiO_2_ and respiratory rate were titrated to maintain a partial pressure of oxygen (PaO_2_) of >95 mmHg and normocapnia. PEEP was maintained at 5 cm H_2_O to minimize de-recruitment and was not increased during the study, as this level of PEEP is known to have no effects on cerebral perfusion pressures (24). Neuromonitoring instrumentation included a partial pressure of tissue oxygenation (PTiO_2_) probe (Licox, Oxford Optronics, Ltd, Oxford, UK) and an intracranial pressure (ICP) monitor, inserted via a mini-craniectomy at the left and right fronto-parietal regions, respectively. CPP was derived using the clinical algorithm: CPP = mean systemic arterial blood pressure (MAP)−ICP, with MAP being measured at the level of the heart.

To avoid self-transfusion from the ovine spleen (25, 26), a ligation of the splenic artery was performed through a skin incision parallel to the distal end of the last rib on the left side followed by blunt dissection through the serratus and oblique abdominal muscles. The splenic artery and vein were identified, the artery ligated followed by injection of adrenaline (0.025 mL of 1:10,000 dilutions) directly into the spleen parenchyma and a 60-s waiting period to allow for spleen contraction and the return of the sequestered blood into the systemic circulation.

### Normovolaemic Anemia Model

Acute normovolaemic anemia was achieved by sequential blood extractions from the indwelling arterial catheter performed simultaneously with isovolaemic saline infusions. The aim was to achieve a 30% reduction of baseline hemoglobin to reproduce the fall in hemoglobin concentration that was achieved in the TRICC trial. The targeted hemoglobin was monitored using cardiac index, blood pressure, systemic vascular resistances, and arterial blood gas sampling to ensure stable and normal lactate levels, every 15 min.

### Protocol for Transeptal Catheterization and Injection of Microspheres

Following anesthesia, a transeptal catheter was inserted into the left atrium (LA) under intracardiac echocardiography (ICE) surveillance. Two 11-Fr Terumo sheaths located in the RIJ allowed for the insertion of the intracardiac ultrasound probe (Acuson AcuNav™ probe, California^®^) and the transeptal catheter (Mullins TS introducer, Medtronic^®^). Echocardiography images were obtained using an Acuson Sequoia C512 scanner (Siemens, CA, USA). Transeptal puncture and insertion of a pigtail catheter into the LA followed previously described methods (27).

### Protocol for Microsphere Injection

Randomly assigned color-coded microspheres (E-Z TRAC; Interactive Medical Technology, Los Angeles, CA, USA) were injected hourly via the LA pigtail catheter using the following protocol. Six different colors (*purple low*, *purple high*, *pink high*, *yellow high*, *coral low*, and *coral high*) were used as per the manufacturer’s recommendations. For each injection, a thoroughly mixed vial of one color of microspheres containing 5 million spheres in 0.8 mL volume was used. This microsphere density is recommended by the manufacturer and does not cause microvascular plugging or occlusion (23). At 30 s following the initiation of the blood withdrawal with a pump rate of 10 mL/min, a fast injection of the microspheres followed by a 10 mL saline flush over 10 s was performed. The withdrawal pump was turned off after 2 min of use, and Tween 80 reagent was used to clear the arterial catheter and dilute the microspheres.

### Aspects of the Injection of Color-Coded Microspheres

Approximately, five million color-coded spheres of one color were injected at each hour with the color of the microspheres randomly assigned at each injection time, to minimize selection biases and to enable the quantification of flow at each defined time points. Each sheep had a different microsphere color injected at defined time points to assess temporal changes in RMBF by anatomical area. As per the manufacturer’s recommendations, the microspheres were maintained at room temperature, were not exposed to sunlight, heat, or vibration, and were mixed manually to minimize foaming and avoid non-uniform concentrations of microspheres throughout each injection.

### Euthanasia and Post-Mortem Tissue Manipulation

After 5 h of continuous monitoring and microsphere injection, sheep were euthanized under non-recovered anesthesia with a bolus injection of 0.5 mL/kg of sodium pentobarbitone. After confirmation of death, the brain was harvested by craniotomy, weighed, and then fixed in 10% formalin for 3 weeks.

### Brain Harvesting Technique

Brains were harvested using a deep incision of the skin and underlying tissue planes between the first and second occipital vertebral body with the intention of sectioning the spinal cord at that level. A round reciprocating saw was used to create a wide bitemporal bone incision. Rectangular bone sections of ~5 cm were made anteriorly to the bitemporal incision to the frontal sinuses. These bone sections were removed with retractors with simultaneous dissection of the dura. Once the brain was fully exposed, the olfactory bulbs, optic chiasm, tentorium, and cranial nerves were sectioned to liberate the brain from the cranium.

### Tissue Sampling Model

**Figure 1:**
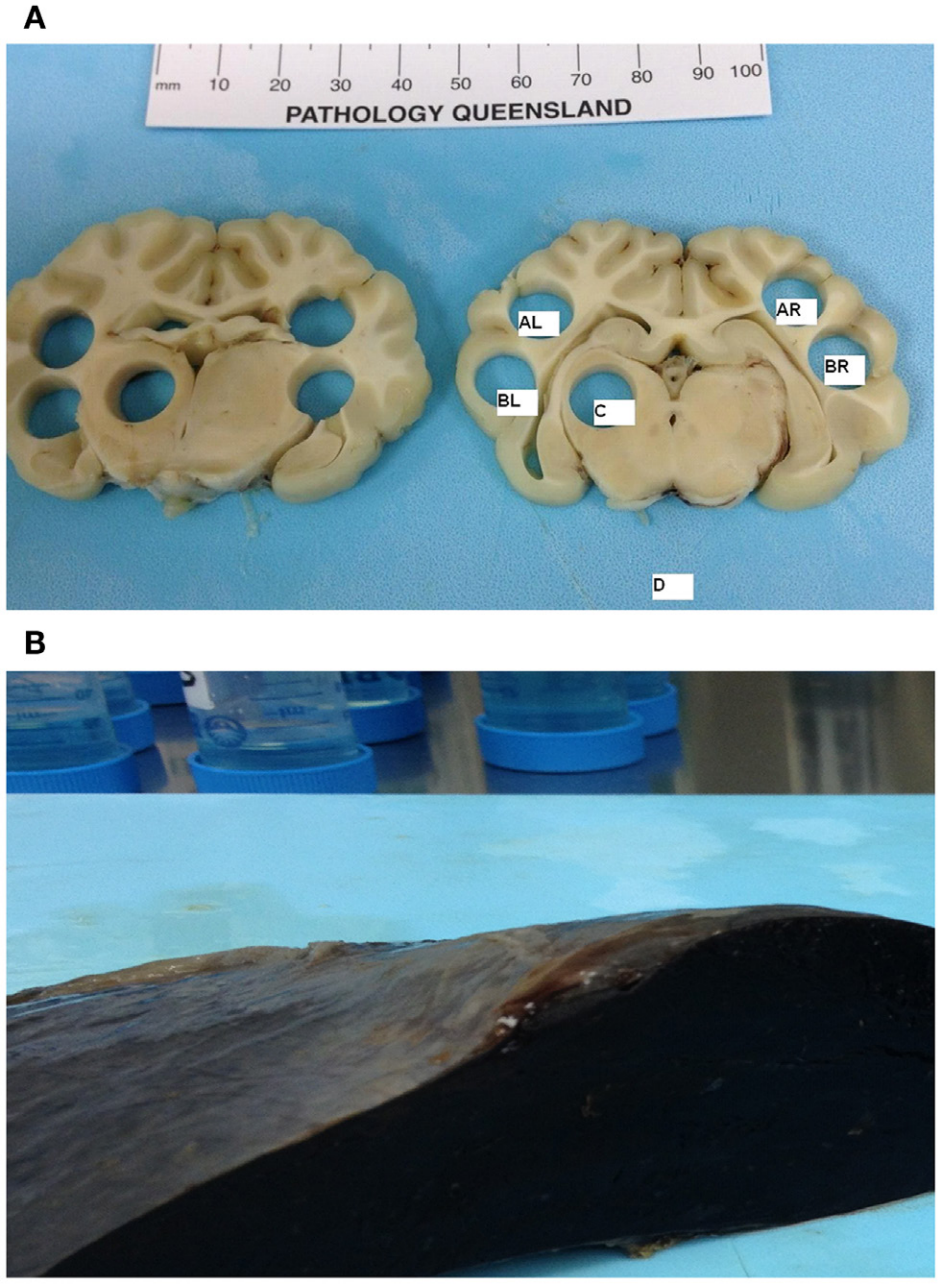
**(A)** Cone post-mortem and post-fixation samples representing the anatomical regions of interest. AL, left parietal; BL, left temporal; C, left-sided basal ganglia; AR, right parietal; BR, right temporal, D, Medulla. **(B)** Post-mortem spleen showing macroscopic evidence of infarct as a result of successful splenic artery ligation.

A novel design for tissue sampling was developed to capture the specific anatomical regions of interest (Figure 1A). After creating 5-mm brain blocks, 0.4 g cone samples were extracted from the hemispheres (AR corresponding to right parietal cortex, BR corresponding to right temporal cortex, AL corresponding to left parietal cortex, and BL corresponding to left temporal cortex), basal ganglia (C), and medulla (D). Adjacent tissue blocks were assigned for both cytometric and histological analyzes. This novel design allowed us to directly quantify axonal damage and RMBF in specific anatomical regions, which could be compared between subjects with different brain sizes.

Samples of skin, gut, kidney, spleen, and heart were harvested to demonstrate the systemic distribution of the microspheres. The spleen was sampled to demonstrate splenic infarct due to successful arterial spleen ligation and the absence of microspheres (Figure 1B).

### Quantification of Microvascular Blood Flow

Cytometric counts of the microspheres were performed using a validated technique (23, 28–30). Cytometric count of microspheres allows the calculation of RMBF in each particular organ through a process that includes the specific microspheres concentration injected into the systemic circulation at each time point and the amount of spheres in a reference sample of arterial blood, known also as the “reference sample of blood”, extracted also at each time point. The reference sample was an arterial blood sample withdrawn at a known rate over a fixed period of time (31). RMBF was the proportion of microspheres trapped in the targeted tissue in relation to the total quantity of spheres per milliliter of blood per minute of the reference sample and was calculated using the following algorithm (32):

RMBF(mL/min/g)=(Total tissue spheres)/[(Tissue weight, g)× (Reference Spheres/mL/min)]

This cytometric analysis was performed at Interactive Medical Technology (IMT), Los Angeles, CA, USA.

### Immunohistochemistry Processing

Immunohistochemistry analysis was performed at the neuropathology laboratory, Royal Brisbane and Women’s Hospital, QLD, Australia, using a Leica Novolink Polymer Detection Systems Kit (Leica Microsystems Pty Ltd., North Ryde, 2113 Australia) as per the manufacturer’s instructions. Paraffin was removed from the sections through a series of xylene immersions and re-hydrations. Antigen retrieval was carried out using Leica BOND ER1 solution. Endogenous peroxidase was neutralized. Sections were incubated with a protein block. The primary antiserum made up in Leica BOND Antibody Diluent was applied to the sections.

### Immunohistochemistry and Hematoxylin–Eosin Scoring and Interpretation

Immunohistochemistry analysis was applied to the sheep brains for the baseline presence and location of APP antibodies. APP antibody staining was used because it is considered to be a very early marker of neuronal damage (33) and this study had a short follow-up period (4 h post intervention). A grading system measuring the presence of APP was developed and structured into three qualitative categories dependent on the severity of injury: *Mild Injury*: focal contusion with APP labeling limited to the site of the injury or focal APP labeling; *Moderate Injury*: a pattern of APP staining greater than one hemisphere, greater than half a hemisphere, or less than half a hemisphere; and *Severe Injury*: the presence of diffuse staining sub-classified as either diffuse vascular injury, diffuse axonal injury with macroscopic hemorrhage, diffuse axonal injury with microscopic hemorrhage/tissue tears, or diffuse axonal injury only (34). For each animal, samples were taken from each brain anatomical region of interest for both cytometric counts of RMBF and immunohistochemistry.

### Statistical Analysis

#### RMBF Analysis

Regional microvascular blood flow was measured at baseline with no anemia (corresponding to time zero – T0) and at four follow-up times following the induction of anemia (T1–T4 corresponding to first to the fourth hour post intervention). A repeated-measure mixed regression model was used to examine differences in RMBF associated with anemia, and a mixed model with a random intercept was used for each sheep to control for repeated results from the same sheep.

To examine the variability in RMBF, all results were plotted using box-plots. We also calculated the percentage of the total variation in the mixed models due to the between-sheep ­variability. A high percentage (above 50%) indicated that the variance in RMBF was due to differences between sheep, whereas a lower percentage indicated that the differences between sheep were small. The analysis was performed used R software version 3.0.2.

## Results

**Figure 2:**
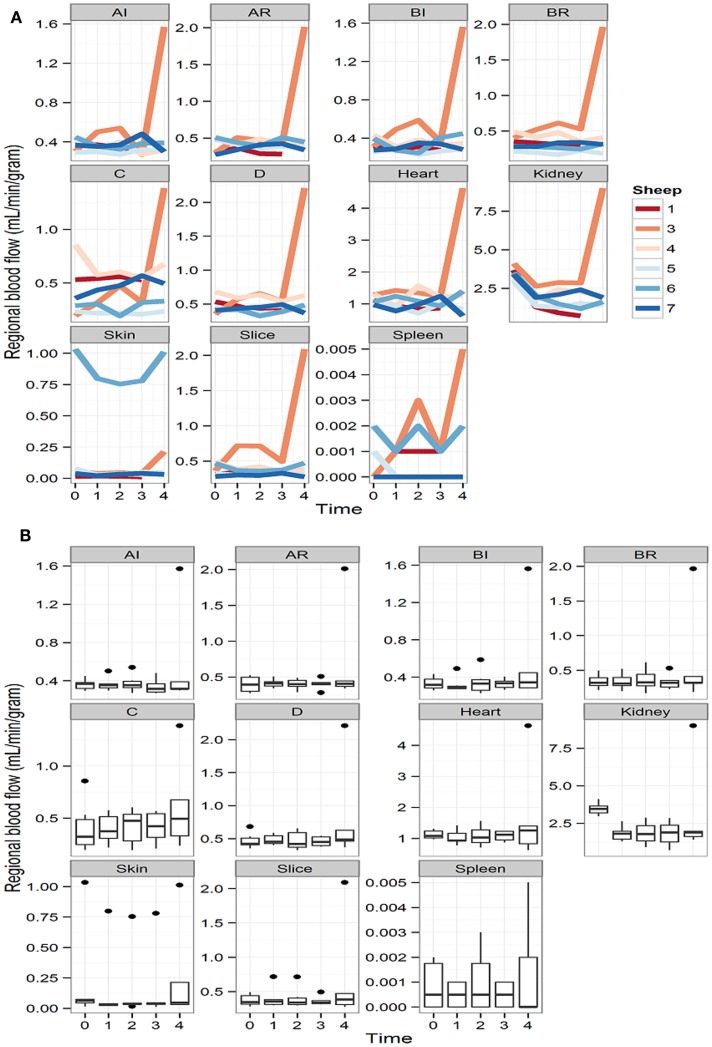
**(A)** Representation of the regional blood flow for each subject over time at each particular region of interest and other extra-cranial tissues. Time is represented as T0 (baseline time or time prior to intervention); T1 (first hour post intervention); T2 (second hour post intervention); T3 (third hour post intervention), and T4 (fourth hour post intervention). **(B)** RMBF means at each time point of all subjects, showing stable, homogeneous, and sustained RMBF during anemia. Time is represented as T0 (baseline time or time prior to intervention); T1 (first hour post intervention); T2 (second hour post intervention); T3 (third hour post intervention); and T4 (fourth hour post intervention).

Despite a fall in hemoglobin to levels between 6 and 7 g/dL (a 30% of the baseline hemoglobin in each animal) with normovolaemia, cerebral RMBF throughout all anatomical areas of interest was maintained in a physiological range after correction to conventional units (mL/100 g/min) during the 4 h of studying time. However, subject number 3 had a significant increase in RMBF at 4 h (T4) with otherwise similar RMBF values at earlier time points (Figure 2A). This was due to the reference blood sample containing significantly fewer microspheres due to a most likely, defective withdrawal pump rate, leading to an overestimation of RMBF.

Box-plots (Figure 2B) show changes in RMBF variability over time. The heights of the boxes and their means are similar, suggesting that the values were maintained within similar ranges. The dots indicate the presence of some outliers.

The effect of anemia on RMBF during the 4 h of study time is presented in Table 1. Anemia decreased the RMBF in the kidneys. The highest ratio of 97% was observed in the skin. The between-sheep variability seen in Figures 2A,B depended mainly on the outlier values of subject 6.

**Table 1:** **Effect of anemia on RMBF by anatomical regions**.

Tissue	Mean	Lower	Upper	*p*-value	Ratio
AI	0.045	−0.166	0.256	0.676	7
BI	0.053	−0.150	0.258	0.609	19
AR	0.075	−0.203	0.354	0.6	3
BR	0.067	−0.186	0.322	0.601	29
C	0.050	−0.148	0.247	0.619	28
D	0.077	−0.221	0.375	0.613	11
Slice	0.082	−0.189	0.353	0.553	26
Skin	−0.042	−0.101	0.016	0.164	97
Spleen	0.000	−0.001	0.001	0.989	42
Kidney	−1.371	−2.505	−0.229	0.0248	30
Heart	0.089	−0.508	0.690	0.769	17

The cardiovascular responses to acute normovolaemic anemia are presented in Table 2. For the majority of animals, the physiological response to acute anemia was a reduction in cardiac output and an increase in the systemic vascular resistances. Vascular resistances were not available for subject 1 due to a calibration error.

**Table 2:** **Cardiovascular and cerebral oximetry response to normovolemic anemia**.

Hours post intervention	T 0	Goal: reduction of 30% baseline Hbl	T1	T2	T3	T4
	
	Baseline time		First hour post intervention	Second hour post intervention	Third hour post intervention	Fourth hour post intervention
**SHEEP 1**						
CO (L)	4.8		4.1	2.7	2.5	2.5
SV02 (%)	75		71	46	55	50
SVR (dynes s/cm/m^2^)						
Hbl (g/dL)	**12.8**		10.0	7.6	7.8	8.1
CPP	100		80	70	65	65
PTiO_2_	17.3		8.2	10	5.4	6.1
**SHEEP 3**						
CO	5		4.5	3.6	3.6	3.7
SV02 (%)	85		75	71	75	76
SVR (dynes s/cm/m^2^)	1445		1682	1865	1817	1886
Hbl	**12.4**		7.8	8.1	8.1	8.1
CPP	90		84	84	75	83
PTiO_2_	2.65		2.89	3.26	4.90	5.21
**SHEEP 4**						
CO	4.3		4.0	4.7	4.2	4.3
SV02 (%)	66		70	69	67	67
SVR (dynes s/cm/m^2^)	1250		1060	1240	1270	1280
Hbl	**10.3**		7.9	8.1	7.9	7.9
CPP	122		118	118	107	106
PTiO_2_	25		43	45	42	44
**SHEEP 5**						
CO	4.1		3.9	3.2	2.7	3.2
SV02 (%)	62		62	55	49	56
SVR (dynes s/cm/m^2^)	2245		2258	2460	2622	2367
Hbl	**9.7**		7.5	6.9	7.1	7.0
CPP	109		110	93	100	80
PTiO_2_	26		16	12	13.6	14.1
**SHEEP 6**						
CO	3.4		3.3	3.7	3.0	3.8
SV02 (%)	77		72	66	66	71
SVR (dynes s/cm/m^2^)	2226		2133	1678	1601	1228
Hbl	**11.0**		6.7	7.5	7.5	7.0
CPP	100		82	93	92	92
PTiO_2_	5.7		2.15	2	1.8	2.1
**SHEEP 7**						
CO	4.0		2.6	3.3	3.9	3.8
SV02 (%)	83		70	71	75	75
SVR (dynes s/cm/m^2^)	1490		3196	2064	1694	1682
Hbl	**10.9**		7.9	7.5	7.5	7.2
CPP	99		98	90	85	85
PTiO_2_	37		5.8	9.5	10.2	7.4

*Bold values represent the baseline hemoglobin as a way to magnify the successful achievement of anaemia maintained in the following cut off time-points*.

**Figure 3:**
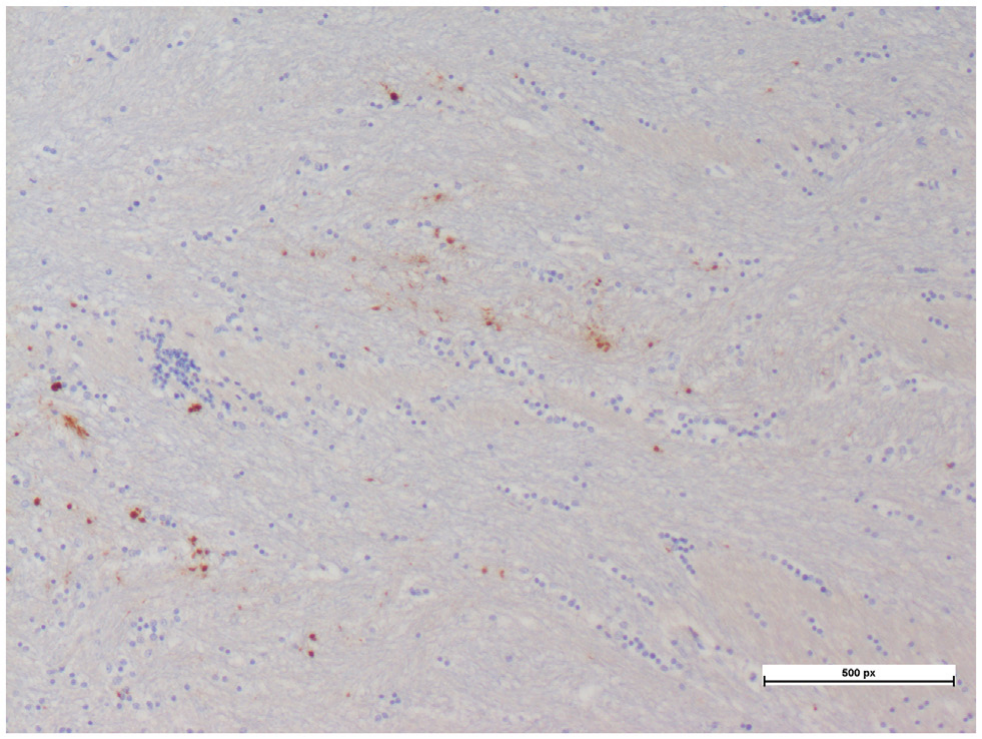
**Petechial hemorrhage with minimal hematoxylin–eosin and APP staining seen in a cerebral tissue sample**.

The cerebral PTiO_2_, CPP, and CO responses to acute anemia are described in Table 2. For the majority of subjects, temporal reductions in cerebral perfusion pressure and cerebral PTiO_2_ were observed during anemia. Despite these changes in regional cerebral oxygenation, both histology and immunohistochemistry were consistent with normal neural tissue, represented by a normal hematoxylin–eosin and APP staining, respectively (Table 3), with only isolated areas of petechial hemorrhage (Figure 3).

**Table 3:** **APP staining score for all anatomical regions of interest and subjects**.

Subject number	Region of interest	H&E staining	APP staining
Sheep 01	AL	Normal	0
	AR	Normal	1
	BL	Normal	0
	BR	Microglial activation	2
	C	Focal hemorrhagic necrosis	2
	D	Normal	0
Sheep 03	AL	Normal	0
	AR	Normal	0
	BL	Normal	0
	BR	Normal	0
	C	Normal	0
	D	Small perivascular petechial hemorrhage	1
Sheep 04	AL	Normal	0
	AR	Normal	0
	BL	Normal	0
	BR	Small perivascular petechial hemorrhage at edge, artifact?	0
	C	Small perivascular petechial hemorrhage at edge, artifact?	0
	D	Normal	0
Sheep 05	AL	Normal	0
	AR	Normal	0
	BL	Normal	0
	BR	Normal	0.5
	C	Normal	0
	D	Normal	0
Sheep 06	AL	Localized focus of acute meningitis and SAH in depth of sulcus	0
	AR	Normal	0
	BL	Normal	0
	BR	Normal	0
	C	Focal hemorrhage near deep gray nuclei	2
	D	Normal	0
Sheep 07	AL	Normal	0
	AR	Normal	0
	BL	Normal	0
	BR	Normal	0
	C	Normal	0
	D	Normal	0

## Discussion

This study demonstrates the maintenance of stable cerebral microcirculation in Merino sheep during and after 4 h of sustained normovolaemic anemia. After the achievement of a 30% reduction of baseline hemoglobin, as in the TRICC trial, despite a reduction in cardiac output and an increase in vascular resistances, RMBF remained unchanged in both intra- and inter-subject comparisons. This observation suggests that despite the cardiovascular impact of anemia, cerebral autoregulation in an otherwise intact brain is perhaps the mechanism involved in the preservation of microcirculation throughout different anatomical regions; however, specific measurement of cerebral autoregulation was not the focus of this study.

Normovolaemic anemia alone in the short term does not appear to have objective effect on brain tissue histo-anatomy. However, cerebral microcirculation and cerebral autoregulation have heterogeneous behaviors that can lead to perfusion mismatch. This characteristic may be magnified in situations where normal perfusion is compromised, such as in head injury and hemorrhagic shock (35, 36) or in pre-morbid conditions affecting the cardiovascular physiology, such as hypertension, vascular diseases, or diabetes. Brain blood flow varies according to the anatomical regional (37) and it remains unclear if this represents a phylogenic remnant or a response to metabolic demands. While many factors have been considered to influence microcirculation (38), the optimal level of hemoglobin during acute head injury remains controversial. In this study, we demonstrate that during acute normovolaemic anemia to levels of hemoglobin of 6 g/dL, RMBF remains normal and unchanged over 4 h. The results of our study are in concordance with the most recent study (12) in acute head injury patients; however, that study was compromised by the concomitant use of erythropoietin and a small sample size.

The experimental strategy used for the direct measurement of RBMF and tissue sampling in this study has proven to be feasible. Quantification of RMBF in specific brain regions allows for intra- and inter-subject comparisons and is a significant improvement on previous approaches using predefined brain thickness sectioning (27). In this study, RMBF in the specific regions of interest shared the same values when a predefined brain slicing technique was used. In addition, with both strategies, RMBF values were within normal ranges.

Limitations of this study are inherent to the experimental nature of the study. Regional microcirculatory blood flow cytometric count in targeted regions of the brain, before and after an insult is not a feasible method in humans. Cerebral cytometric count of microspheres is mainly an experimental concept although it shares the same physical principle of flow cytometry extensively applied in diagnostic medicine (39). Another limitation of this study refers to the short study time; however, the primary intention of this study design was to focus on the direct effect of acute normovolemic anemia in cerebral RMBF at specific anatomical regions of interest, not the long-term effects of anemia in cerebral physiology, let alone neurological outcomes. Finally, this study was applied in healthy subjects as the investigators intended to exclude all possible confounders to the direct effect of short-term anemia in cerebral microcirculatory dynamics. It is plausible to presuppose that the same degree of anaemia in pre-morbid subjects could have led to different RMBF distribution; however, this was not the main aim of the study; but to establish the direct effect of anemia in cerebral microcirculation as a preliminary model to compare with future head injury models.

This study achieved predefined aims and followed a rigorous methodology, demonstrating that short-term normovolemic anemia does not impair cerebral RMBF at specific anatomical regions, a model not feasible in the clinical arena, as an adjunct data to future head injury studies.

## Conclusion

Acute normovolaemic anemia replicating a restrictive transfusion strategy in ovine models without head injury does not impair cerebral microcirculation or induce axonal damage.

## Author Contributions

Primary roles: JB, Study design, surgical procedures and data collection, data interpretation, and manuscript preparation. KC, Histopathology analysis. KD, Laboratory support and manuscript preparation. SD, surgical procedures and data collection. DP and OR, intracardiac echography and transeptal catheterization. LG, manuscript preparation. AB, statistical analysis. JP, RB and JF, data interpretation and manuscript preparation.

## Conflict of Interest Statement

The authors declare that the research was conducted in the absence of any commercial or financial relationships that could be construed as a potential conflict of interest.
